# Quality Detection of Litchi Stored in Different Environments Using an Electronic Nose

**DOI:** 10.3390/s16060852

**Published:** 2016-06-09

**Authors:** Sai Xu, Enli Lü, Huazhong Lu, Zhiyan Zhou, Yu Wang, Jing Yang, Yajuan Wang

**Affiliations:** 1 Guangdong Engineering Research Center of Agricultural Product Cold Chain Logistics, South China Agricultural University, Guangzhou 510642, China; xusai1991@sina.cn (S.X.); huazlu@scau.edu.cn (H.L.); yu-wang@scau.edu.cn (Y.W.); ziji90821@sina.com (J.Y.); wangyaj90@sina.com (Y.J.W.); 2 College of Engineering, South China Agricultural University, Guangzhou 510642, China; zyzhou@scau.edu.cn

**Keywords:** electronic nose, litchi, quality detection, storage environment, pattern recognition

## Abstract

The purpose of this paper was to explore the utility of an electronic nose to detect the quality of litchi fruit stored in different environments. In this study, a PEN3 electronic nose was adopted to test the storage time and hardness of litchi that were stored in three different types of environment (room temperature, refrigerator and controlled-atmosphere). After acquiring data about the hardness of the sample and from the electronic nose, linear discriminant analysis (LDA), canonical correlation analysis (CCA), BP neural network (BPNN) and BP neural network-partial least squares regression (BPNN-PLSR), were employed for data processing. The experimental results showed that the hardness of litchi fruits stored in all three environments decreased during storage. The litchi stored at room temperature had the fastest rate of decrease in hardness, followed by those stored in a refrigerator environment and under a controlled-atmosphere. LDA has a poor ability to classify the storage time of the three environments in which litchi was stored. BPNN can effectively recognize the storage time of litchi stored in a refrigerator and a controlled-atmosphere environment. However, the BPNN classification of the effect of room temperature storage on litchi was poor. CCA results show a significant correlation between electronic nose data and hardness data under the room temperature, and the correlation is more obvious for those under the refrigerator environment and controlled-atmosphere environment. The BPNN-PLSR can effectively predict the hardness of litchi under refrigerator storage conditions and a controlled-atmosphere environment. However, the BPNN-PLSR prediction of the effect of room temperature storage on litchi and global environment storage on litchi were poor. Thus, this experiment proved that an electronic nose can detect the quality of litchi under refrigeratored storage and a controlled-atmosphere environment. These results provide a useful reference for future studies on nondestructive and intelligent monitoring of fruit quality.

## 1. Introduction

As a typical subtropical fruit, litchi (*Litchi chinensis*) is popular all around the world. However, its freshness, flavor and nutritive value decline quickly during the transportation and storage process. From a statistical point of view, the loss of litchi caused by natural decay accounts for more than 20% of the total amount of crop loss each year in China [1]. Investigation and research show that when the brown area of the surface of a litchi occupies 1/4 of the whole surface area, the flavor of the litchi will be seriously influenced, and the total soluble solid content will be decreased, resulting in a reduction in the market value [2]. However, accurate and rapid food quality detection can provide a scientific reference for quality grading, shelf management and consumption, thereby decreasing the losses caused by decay, which is vitally important to the perishable postharvest litchi.

Two commonly used quality detection methods already exist for litchi, namely, the sensory evaluation method [3,4] and the physicochemical indexes detection method [5,6]. The sensory evaluation method evaluates the quality of litchi via human vision, taste and smell. This method is time-consuming, destructive and laborious and is highly influenced by subjective factors. In addition, the physicochemical indexes detection method judges the quality of a litchi by detecting its physical indexes and chemical indexes. It is an intuitive and effective quality detection method, without interference from subjective factors, but the physicochemical indexes detection method suffers from being a complex operation (including the extraction of fruit juice and preparation of testing reagents, *etc.*), with low detection speed (it takes a long time to acquire the physicochemical indexes of the samples one at a time), a high level of effort (a tester must perform the whole testing process) and the need to damage the food itself, so this method cannot meet the need for non-destructive and intelligent detection (automated sampling and analysis to obtain the information for a sample without damage). That is to say, these two commonly used litchi quality detection methods cannot be applied in a massive litchi quality detection program during a practical period of massive litchi storage, and will damage litchi fruits. Therefore, it is reasonable to explore nondestructive and intelligent detection methods for the problem of detection of litchi quality, a problem which has not yet been solved.

Two existing nondestructive and intelligent food quality detection methods are the near-infrared spectroscopy method [7,8] and the electronic nose detection method [9,10]. The near-infrared spectroscopy method can quickly acquire external and internal quality information of samples. However, due to mutual shadowing among litchi fruit during storage, the difficulty of the overall detection is increased greatly. As a method simulating the biological sense of smell, the electronic nose is capable of effectively detecting both simple and complex volatiles, which avoids the influence of subjective factors and field angle. Compared with other detection methods that exist, the electronic nose is more suitable and provides a new means for the quality detection of litchi. Currently, the electronic nose technology has been applied to many fields of research for food quality detection, such as fruits [11,12], vegetables [13,14], meats [15,16] and nuts [17,18], and all of these studies have shown the feasibility of applying an electronic nose to detect food quality. However, research on the application of an electronic nose for the quality detection of litchi during storage has not yet been reported in any publication. The realization of litchi quality detection via an electronic nose can also provide a nondestructive and intelligent way for mass real-time monitoring of litchi quality which cannot be achieved by other existing food quality detection methods. In addition, a set of appropriate recognition algorithms (including feature extraction and pattern recognition) is important for intelligent detection. This research work also provides a reference for selecting recognition algorithms for the intelligent detection of litchi quality.

Our preliminary study found a high correlation between the electronic nose response and the physicochemical indexes for litchi [19]. However, whether the electronic nose can predict the quality of litchi requires further research. To explore the feasibility of using electronic nose technology for the quality detection of litchi, this study acquired both electronic nose data and physicochemical indexes of litchi stored in three different environments (room temperature, refrigerator and controlled-atmosphere) for different storage times. Then, the four different methods of linear discriminant analysis (LDA), canonical correlation analysis (CCA), back-propagation neural network (BPNN) and back-propagation neural network-partial least squares regression (BPNN-PLSR) were employed for data analysis. The main objectives of this study were the following: (1) validating the feasibility of the electronic nose for detecting and monitoring the change in quality of litchi stored in different environments; and (2) predicting the physicochemical index of litchi stored in different environments based on the electronic nose data.

## 2. Materials and Methods

### 2.1. Materials and Grouping

The experimental litchi samples (Guiwei, maturity grade is 8 to 9) were harvested from the Conghua litchi orchard, in Guangzhou (Guangdong, China), in July 2015. All litchi samples were transported to South China Agricultural University (SCAU, Guangzhou, Guangdong, China) within 2 h after harvesting. After removing the branches and leaves, undamaged litchi fruits of uniform size were selected. Then, they were randomly divided into three groups: the room temperature environment storage group, refrigerator environment storage group and controlled-atmosphere storage group. Before storage, litchies of the refrigerator environment and controlled-atmosphere storage groups were precooled [20] for 5 min using ice water (4 to 5 °C). Litchies for room temperature storage were not precooled considering that the decay rate of litchi will increase when transferring litchi from a cool to a warm environment [21]. Perforated polyethylene bags with a perforation ratio of 5% and a size of 300 mm × 200 mm × 0.05 mm were used to pack litchi samples, with 30 litchies per bag. All bags were bought from the SCAU market and manually punched with a hole punch. After that, all of the packed samples were put into plastic crates stored in the different environments. The room temperature storage environment was 25 °C; the refrigerator storage environment was 3 to 5 °C; the controlled-atmosphere storage environment was 3 to 5 °C at 90% to 95% relative humidity, 3% to 6% oxygen content.

Due to its fast decay, litchi stored at room temperature usually loses its commodity value within 5 days after harvest [2]. Thus, litchies in the room temperature environment were tested at 0, 1, 2, 3 and 4 days after harvest in this experiment. Because the refrigerator environment and controlled-atmosphere environment can extend the freshness lifetime of litchi [22], litchies in the refrigerator environment and controlled-atmosphere environment were tested at 0, 2, 4, 6 and 8 days after harvest. Thus, in this experiment, we tested electronic nose data and hardness for litchi during storage. Twenty samples from each storage environment were prepared for each test, so in total, this study obtained 300 sets of electronic nose data and 300 physicochemical indexes.

### 2.2. Experimental Storage Equipment

Air-conditioning (KFR-72LW, Gree Ltd., Zhuhai, China), a refrigerator (GX, Guangxiang Ltd., Guangzhou, China) and our laboratory-developed controlled-atmosphere storage platform [23] were used as the equipment for room temperature storage, refrigerator storage and controlled-atmosphere storage, respectively. The inside environment of the laboratory-developed controlled-atmosphere storage platform can be adjusted according to the requirements.

### 2.3. Electronic Nose Detection

A PEN3 portable electronic nose (Airsense Ltd., Schwerin, Germany) was employed in these experiments. This electronic nose system is mainly composed of sampling and cleaning channels, and a sensor array and pattern recognition unit. The sensor array is composed of 10 metal-oxide semiconductor-type chemical sensors, and their features are presented in Table 1. Each sample included one litchi fruit, and was enclosed in a 200-mL beaker, then sealed with plastic wrap for 0.5 h, and the electronic nose was used to sample its headspace. Beakers were washed using an ultrasonic cleaning instrument and cooled in the shade, and no peculiar aroma was detected. Before sampling, zero gas (room air that had been filtered through standard activated carbon) was pumped into the cleaning channel to normalize the gas sensors. The operating parameters of the electronic nose were setting at sampling interval of 1 s; flush time of 60 s; zero point trim time of 10 s; measurement time of 80 s; presampling time of 5 s; and injection flow of 300 mL/min.

### 2.4. Physicochemical Index Measurement

There are some studies focused on physicochemical index prediction of fruit such as soluble solids content [24], titratable acidity [25] and pH value [26], *etc.*, based on electronic nose data. The hardness is an important quality index of fruit which is directly related to the taste [27], and often changes with the degree of freshness of fruit [28]. Some studies have focused on the correlation between electronic nose data and food hardness [29,30,31], and their results indicate the high correlation between electronic nose data and food hardness. However, whether the hardness of litchi can be predicted by an electronic nose has not yet been reported. Thus, this experiment tested the hardness as a quality index of litchi. After sampling by the electronic nose, the peel of litchi samples was removed immediately to measure the hardness of the pulp. The hardness measurement method applied in this experiment was the one proposed by Luo [32], and a portable fruit hardness tester (GY, Aidebao Ltd., Leqing, China) was used in these experiments. The average value of the hardness value for each sample was obtained from triplicate measurements.

### 2.5. Data Analysis Methods

#### 2.5.1. LDA

LDA is one of the most commonly used classification procedures. The method maximizes the variance between categories and minimizes the variance within each single category. This method renders a certain number of orthogonal linear discriminant functions which equals the number of categories minus one [33].

#### 2.5.2. CCA

CCA can focus on the relationship between two groups of variables [34]. The CCA analysis process involves building a linear combination for each group of variables based on the total variation of their original data matrixes, finding the most relevant aggregate variable (canonical correlation variable) from the linear combinations, and then, revealing the related properties of the two groups of variables via the canonical correlation variable. The correlation coefficient (r) and significance (sig) are two important parameters to judge the degree of correlation when using CCA for analysis. The definitional domain of r is [–1, 1]. The positive r value means positive correlation, and the negative value means a negative correlation. If the value of |r| is larger, the correlation is more significant. When the sig is less than 0.05, it means the correlation is significant. The smaller sig is, the more significant the correlation is. The “canoncorr” function tool of Matlab was used to perform the CCA analysis in this research.

#### 2.5.3. BPNN

BPNN is one of the most widely employed ANN models [35]. It can be described as a non-linear projection between the input vectors and output vectors. A typical BPNN structure has three parts: one input layer, one hidden layer, and one output layer. In the process of training BPNN for analysis, the weights and threshold values of each layer are revised constantly, and this training lasts until the difference between the expected outputs and actual outputs is limited to a preliminary range or the scheduled training times are achieved.

#### 2.5.4. PLSR

The partial least-squares regression (PLSR) is a technique used with data that contains correlated predictor variables. This technique constructs new predictor variables, known as components, as linear combinations of the original predictor variables. PLSR constructs these components while considering the observed response values, leading to a parsimonious model with reliable predictive power [36].

## 3. Results

### 3.1. Hardness Change of Litchi Stored in Three Environments

**Figure 1:**
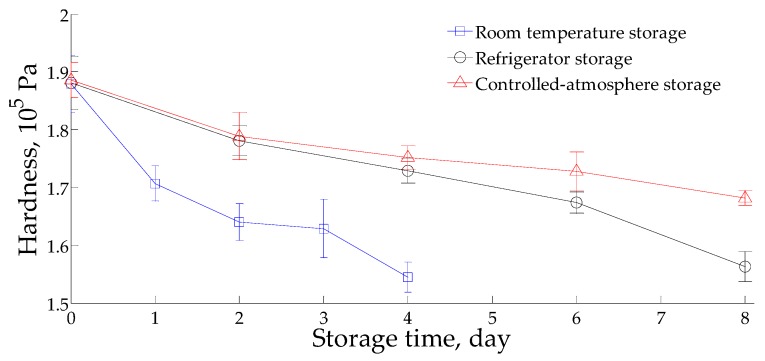
Change in hardness of litchi stored in the three environments.

The change in hardness for the stored litchi in the three storage environments is shown in Figure 1. During storage, the hardness of all of the litchies decreased. In comparison, the litchi stored at room temperature had the fastest rate of decrease, followed by those in the refrigerator environment and controlled-atmosphere environment.

### 3.2. Change in Sensor Response Signal for Litchi Stored in Three Environments

To investigate the change in sensor response for litchi in different storage environments, the sensor response values in the 80th second were applied as each sample’s feature value. The average value of 20 samples was used as the sensor response value of each storage group (room temperature environment storage group, refrigerator environment storage group and controlled-atmosphere storage group) at a given storage time. The variation in the sensor response value for each storage group was measured on the 0th, 2nd and 4th day as presented in Table 2. On day 0, the differences in sensor response values between each storage group were small. However, after the 0th day, the electronic nose response value for the room temperature storage group changed greatly. In contrast, those of the refrigerator environment storage group and controlled-atmosphere storage group showed a slight change. With the increase in storage time, for litchi stored at room temperature, the response values of R1, R3, R5 and R10 were gradual; however, the response values of other sensors increased gradually. With an increase in storage time, for litchi stored in the refrigerator environment, the response values of R1, R3, R4, R5, R7 and R10 decreased gradually, and the response values of R8 and R10 first increased and then decreased, while the response values of the other sensors increased gradually. With an increased storage time for litchi stored in a controlled-atmosphere environment, the response values of R1, R3, R4 and R5 decreased gradually, and the response values of R7, R8 and R10 first increased and then decreased; in contrast, the response values of the other sensors increased gradually. Thus, according to the electronic nose response, the laws governing the change in volatiles in litchi stored in the three environments were different, which also proved the feasibility of using the electronic nose for detection of the changes in volatiles of litchi stored in different environments.

### 3.3. LDA for Storage Time Classification

In this study, three types of feature values were applied for pattern recognition: (1) the maximum value [37], the maximum value of a complete response value; (2) the average of the differential value [38], the average value of the differential of the electronic nose response curve for each sampling time; and (3) the 80th s value [39,40], the 80th s of each response curve. After feature extraction, those three feature values were tested by LDA. 

**Figure 2:**
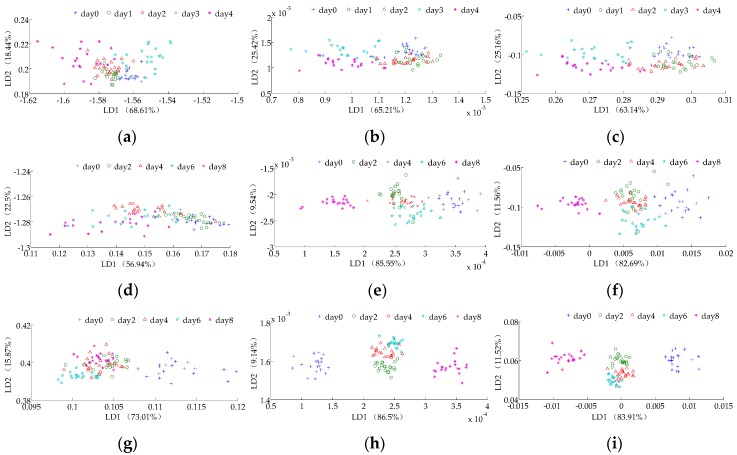
LDA analysis results of litchies stored in different environments based on (**a**,**d**,**g**) the maximum value; (**b**,**e**,**h**) the average of the differential values; (**c**,**f**,**i**) the 80th s value. (**a**–**c**) are the LDA analysis results of the room temperature storage group (**d**), (**e**) and (**f**) are the LDA analysis results of the refrigerator environment storage group; (**g**–**i**) are the LDA analysis results of the controlled-atmosphere environment storage group. Note: LD1 is the contribution rate of the first linear discriminant factor, LD2 is the contribution rate of the second linear discriminant factor.

The LDA analysis results of the storage time for the litchi stored at room temperature (from the 0th to the 4th day) based on the maximum values, the average of differential values and the 80th s values are shown in Figure 2a–c, respectively. When the maximum values were applied as the feature value of LDA analysis of the storage time for litchi stored in the room temperature environment (Figure 2a), all storage times overlap with others and cannot be classified. When the average of differential values was applied as the feature value for LDA analysis of litchi stored at room temperature (Figure 2b), none of the storage times can be classified, and the classification effect is poor. When the 80th s values were applied as the feature value for LDA analysis of storage time for the litchi stored at room temperature (Figure 2c), only the 4th day can be classified. Other storage times overlap with those of the other days, which shows that the classification effect is also unsatisfactory.

The LDA analysis results of the storage time for litchi stored in a refrigerator (from the 0th to the 8th day) based on the maximum values, the average of differential values and the 80th s values are shown in Figure 2d–f, respectively. When the maximum values were applied as the feature value for LDA analysis for the storage time for litchi stored in a refrigerator environment (Figure 2d), all storage times overlap with the others and cannot be classified. When the average of differential values was applied as the feature value for LDA analysis of the storage times for the litchies stored in the refrigerated environment (Figure 2e), none of the storage times can be classified, except those on the 0th and 5th day that were not overlapped by others. When the 80th s values were applied as the feature value for LDA analysis for the refrigerated environment storage group (Figure 2f), only the 0th and 5th day can be classified via this method. However, the 2nd, 4th, 6th and 8th day cannot be classified.

The LDA analysis results of the storage time for litchi stored under a controlled-atmosphere (from the 0th to the 8th day) based on the maximum values, the average of differential values and the 80th s values are shown in Figure 2g–i, respectively. When the maximum values were applied as the feature value for LDA analysis (Figure 2g), all of the storage times were overlapped by others except the 4th day that can be classified. When the average of the differential values was applied as the feature value for LDA analysis (Figure 2h), the 0th, 2nd and 8th days were not overlapped by any others and thus can be classified. However, the 4th and 6th cannot be classified. The data on the 4th and 6th days were overlapping each other and cannot be classified. When the 80th s values were applied as the feature value for LDA analysis (Figure 2h), the 0th, 2nd, and 8th days can be classified. However, the 4th day and 6th day overlap each other and cannot be classified.

Based on the analysis results above, the storage time classification effect of litchi stored in the controlled-atmosphere environment is the best, followed the by refrigerator environment and room temperature environment. In addition, according to the classification effect based on different feature values, the classification effects based on the average of differential values and the 80th s values were clearly better than the maximum values. The study of Hu *et al.* found that the total discriminant factor contribution is higher and that the classification effect is better [41]. Thus, we selected average of the differential values (which has the highest total contribution of LD1 and LD2) as the feature value for the next analysis in this experiment.

### 3.4. BPNN for Storage Time Classification

Because the classification effect of the linear identification method (LDA) for storage time is not satisfactory, this study applied a nonlinear identification method (BPNN) for further classification. There are 20 samples tested at five storage times for each environment group. The experiment selected 15 samples randomly from 20 samples of each storage time for each environment group as a training set. The remaining five samples from the 20 samples are the test set. Thus, the training set includes 75 samples in total, and the test set includes 25 samples in total. We ranked the expected output of five storage times for each storage environment from small to large. They are (1, 0, 0, 0, 0), (0, 1, 0, 0, 0), (0, 0, 1, 0, 0), (0, 0, 0, 1, 0), (0, 0, 0, 0, 1). After repeated training, the model parameters and recognition accuracy are given in Table 3. The classification accuracy of training sets of the room temperature environment storage group, the refrigerator environment storage group and the controlled-atmosphere storage group are 89.33%, 100% and 100%, respectively. The classification accuracy of test sets of the room temperature environment storage group, the refrigerator environment storage group and the controlled-atmosphere storage group are 52%, 88% and 96%, respectively. Thus, the storage time of the refrigerator environment and the controlled-atmosphere stored litchi can be classified effectively via the electronic nose. However, the storage time classification effect of litchi stored in the room temperature environment is still poor.

### 3.5. CCA for Correlation Analysis between Electronic Nose Data and Hardness of Litchi

**Figure 3:**
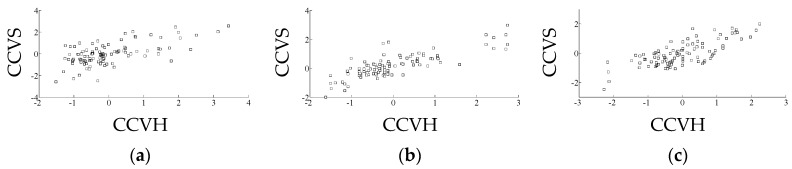
The CCA plot of electronic nose data and hardness of litchi stored in (**a**) the room temperature environment; (**b**) the refrigerator environment; and (**c**) the controlled-atmosphere environment. Note: The CCVS is the canonical correlation variable of the sensor response data, the CCVH is the canonical correlation variable of the hardness values.

In this experiment, the CCA was applied to test the correlation between electronic nose data and hardness of litchi stored in three environments, and the “canoncorr” function command of Matlab was used to perform the CCA analysis. The CCA plots (Figure 3) illustrate a strong linear correlation between the canonical correlation variable of the sensor response data and the canonical correlation variable of the hardness values, indicating that the electronic nose response were linearly correlated with hardness values of litchi stored in the three environments. The |r| values between the electronic nose data and the hardness of litchi stored in the room temperature environment, the refrigerator environment and the controlled-atmosphere environment are 0.5919 (sig = 1.29 × 10^−2^), 0.7104 (sig = 8.85 × 10^−3^) and 0.7439 (sig = 8.04 × 10^−3^), respectively. Thus, CCA results show a significant correlation (|r| > 0.5, sig < 0.05) between electronic nose data and hardness data under the room temperature, and the correlation is more obvious (|r| > 0.7, sig < 0.01) for those under the refrigerator environment and controlled-atmosphere environment.

### 3.6. BPNN-PLSR for Hardness Prediction

To study the prediction effect of the physicochemical index of the litchi during its storage based on the electronic nose, this study applied BPNN-PLSR for hardness prediction of litchi stored in the three different environments. We used the same training set and test set as the BPNN analysis for storage time classification in this research. In addition, a global model (mixed litchi samples stored in all three environments were used for model training) was also be built instead of the three separate models to try to simplify the prediction process. We chose 75 samples randomly from each environment storage group as the training set of global environment storage group, and 25 samples were randomly selected from the remaining samples of each environment storage group as the test set of the global environment storage group. The expected outputs are the actual hardness of the litchi. The process of BPNN-PLSR for hardness prediction is: (1) build a BPNN model for hardness prediction; (2) obtain the predicted hardness value via the BPNN prediction model; and (3) apply PLSR for fitting the predicted hardness value and the actual hardness value and determining litchi’s physicochemical index prediction effect according to the PLSR fitting result. The training parameters of the BPNN prediction model after repeated training for three storage environments are shown in Table 4.

**Figure 4:**
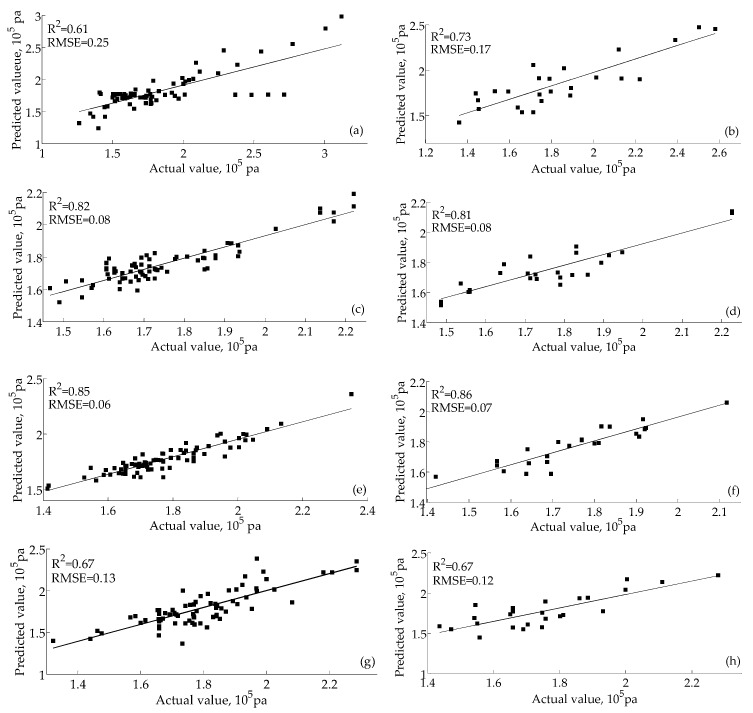
Hardness prediction results of litchi stored in different environments based on BPNN-PLSR. (**a**) Training set of litchi stored at room temperature; (**b**) test set of room temperature storage group; (**c**) training set of the refrigerator environment storage group; (**d**) test set of refrigerator environment storage group; (**e**) training set of the controlled-atmosphere environment storage group; (**f**) test set of the controlled-atmosphere environment storage group; (**g**) training set of the global environment storage group; (**f**) test set of the global environment storage group.

When PLSR is used for regression fitting, the fitting correlation coefficient (R^2^) and the root mean square error (RMSE) are two important indexes for judging the correlation effect. It is noted that according to [37], if the R^2^ value is greater than 0.8, this demonstrates that the prediction value has a high correlation with the actual value. If the R^2^ is closer or further from 1, the correlation is higher or lower, respectively. However, if the R^2^ is lower than 0.8, this demonstrates that the prediction value has a low correlation with the actual value and that the prediction is unfeasible. In addition, the closer the RSME value is to 0, the better the prediction effect. The BPNN-PLSR hardness prediction effects are illustrated in Figure 4.

The BPNN-PLSR hardness prediction results of the training set and the test set for the room temperature environment storage group are shown in Figure 4a,b, respectively. For the BPNN-PLSR hardness prediction results of the training set of the room temperature environment stored litchi, the R^2^ and RMSE values are 0.61 and 0.25, respectively. For the BPNN-PLSR hardness prediction results for the test set of the room temperature environment storage group, the R^2^ and RMSE are 0.73 and 0.17, respectively. Thus, the quality-prediction effect of the electronic nose for the room temperature environment storage group is weak (the R^2^ values of the training set and test set are less than 0.8). The BPNN-PLSR hardness prediction results of the training set and the test set of the refrigerator environment storage group are shown in Figure 4c,d, respectively. For the BPNN-PLSR hardness prediction results of the training set of the refrigerator environment storage group, the R^2^ and RMSE values are 0.82 and 0.08, respectively. For the BPNN-PLSR hardness prediction results of the test set of the refrigerator environment storage group, the R^2^ and RMSE values are 0.81 and 0.08, respectively. Thus, it is feasible to apply the electronic nose for the prediction of the quality of litchi stored in a refrigerator environment (the R^2^ values of the training set and test set are greater than 0.8). The BPNN-PLSR hardness prediction results of the training set and the test set of the controlled-atmosphere environment storage group are shown in Figure 4e,f, respectively. For the BPNN-PLSR hardness prediction results of the training set of the controlled-atmosphere environment storage group, the values if R^2^ and RMSE are 0.85 and 0.06, respectively. For the BPNN-PLSR hardness prediction results of the test set of the controlled-atmosphere environment storage group, the values of R^2^ and RMSE are 0.86 and 0.07, respectively. Thus, the quality prediction effect of the electronic nose for litchi stored in a controlled-atmosphere environment is good (the R^2^ values of the training set and test set are not less than 0.85). Thus, the electronic nose can effectively predict the hardness of stored litchi in a controlled-atmosphere environment and refrigerator environment. However, the prediction effect of hardness at room temperature is poor. The BPNN-PLSR hardness prediction results of the training set and the test set for the global group are shown in Figure 4g,h, respectively. For the BPNN-PLSR hardness prediction results of the training set of the global environment stored litchi, the R^2^ and RMSE values are 0.67 and 0.13, respectively. For the BPNN-PLSR hardness prediction results for the test set of the global environment storage litchi, the R^2^ and RMSE are 0.67 and 0.12, respectively. Thus, the quality-prediction effect of the electronic nose for the room temperature environment storage group is weak (the R^2^ values of the training set and test set are less than 0.8).

## 4. Discussion

This experiment explored the feasibility of using an electronic nose for the detection of the quality litchi fruits stored in different environments (a room temperature environment, refrigerator environment and controlled-atmosphere environment). The experimental results show that the electronic nose can effectively recognize the storage time and hardness of litchi stored in a refrigerator environment and controlled-atmosphere environment. However, the storage time and hardness detection effect of litchi stored in a room temperature environment based on the electronic nose is poor.

The hardness of litchi in the three environments gradually decreased with increasing storage time. The hardness of the litchi stored in the room temperature environment has the fastest rate of decrease, followed by the litchi stored in the refrigerator environment and controlled-atmosphere environment. Research by Yang *et al.* [41] showed that the hardness of litchi usually decreases gradually during storage due to the influence of respiration and enzymatic degradation of the cell wall. The decrease in hardness is important in demonstrating the ripening and aging of the litchi. Thus, compared with a room temperature environment, the controlled-atmosphere environment and refrigerator environment can better inhibit the rate of respiration intensity and the formation of enzymes that degrade the cell wall and can slow the litchi aging process. The research results of this study showed that among the three storage environments, the storage effect of the controlled-atmosphere environment is the best, followed by the refrigerator environment and room temperature environment.

LDA is poor for storage time classification of litchi stored in the three environments. However, BPNN is useful for storage time classification of litchi stored in the refrigerator and controlled-atmosphere environments. The classification effect of BPNN is better than LDA for storage time classification of litchi. The reason is that LDA is a linear classification method, the classification performance of LDA is based on the sample’s distribution in two-dimensional space. However, BPNN is a nonlinear classification method that can achieve arbitrary nonlinear mapping from input to output. BPNN displays good performance when applied to functional mapping, functional approximation and self-adaption. Thus, the BPNN is better than LDA for nonlinear problems, such as litchi’s quality classification. LDA is used to preliminarily observe the classification effect and the distribution of electronic nose response data points.

The electronic nose can effectively recognize quality information for litchi stored in a refrigerator environment or controlled-atmosphere environment. However, the quality information reorganization effect of litchi stored in a room temperature environment and global environment are poor. Research by Cai *et al.* [42] indicated the differences of volatile compositions and relative contents of frozen and not frozen litchi, which tallied with the results of the sensors’ response value changes for litchi stored in the three different environments. As we know, the electronic nose is an instrument for detection according to a sample’s volatiles. However, not all volatiles in a sample have great contributions to the electronic nose detection, and some of them may disturb the classification and recognition. Thus, future research can use gas chromatography-mass spectrometry (GC-MS) to explore in detail the volatile changes in litchi stored in different environments. To further improve the quality detection accuracy of litchi via the electronic nose, several gas sensors sensitive to the volatiles (which have a remarkable variation trend during storage according to the GC-MS results) can be selected and combined with a suitable recognition algorithm to set up a specialized recognition device for the intelligent detection of litchi quality.

During the practical process of storing litchi, from the transportation after harvest to the market, a refrigerator environment and controlled-atmosphere environment are the main means of storage. Storage in a room temperature environment usually only occurs in the sales of small quantities of litchi. These experimental results show that it is feasible to use an electronic nose combined with the BPNN analysis method for quality detection of litchi stored in refrigerator and controlled-atmosphere environments.

## 5. Conclusions

This experiment applied electronic nose technology for storage time classification and hardness prediction of litchi in different environments (room temperature environment, refrigerator environment and controlled-atmosphere environment). The electronic nose can effectively detect the quality of litchi stored in a refrigerator environment and controlled- atmosphere environment. The quality detection effect of litchi stored in a room temperature environment is poor. The details are as follows:

(1) The hardness of litchi decreases with an increase in storage time. However, the decrease rate in order from fast to slow is room temperature, in a refrigerator environment and in a controlled-atmosphere environment.
(2) The LDA classification effects of the three litchi storage environments on storage time were poor. Comparatively, the classification effect of litchi in a controlled-atmosphere environment is the best, followed by those in the refrigerator environment and room temperature environment.
(3) According to the LDA classification effect, by using the feature extraction methods such as the maximum value method, average of differential value method and the 80th s value method, the classification effect is the best when using the average of differential value method.
(4) BPNN can effectively recognize the storage time of litchi stored in a refrigerator and controlled-atmosphere environment, but its recognition of litchi stored in a room temperature environment is poor. The classification accuracy of the training sets of the room temperature environment storage group, the refrigerator environment storage group and the controlled-atmosphere storage group are 89.33%, 100% and 100%, respectively. The classification accuracy of the test sets of the room temperature environment storage group, the refrigerator environment storage group and the controlled-atmosphere storage group are 52%, 88% and 96%, respectively.
(5) CCA results show a significant correlation between electronic nose data and hardness data under room temperature, and the correlation is more obvious for those under the refrigerator environment and controlled-atmosphere environment. The BPNN-PLSR prediction effect of hardness for litchi in either a refrigerated or controlled-atmosphere environment is good, but it is poor for that in a room temperature environment and in a global environment.
(6) According to the research results of this study, among the three storage environments, the storage effect of the controlled-atmosphere environment is the best, followed by those of the refrigerator environment and room temperature environment.

## Figures and Tables

**Table 1:** Response features of the sensor array.

Number in Array	Sensor Name	Object Substances for Sensing	Threshold Value (mL·m^−3^)
R1	W1C	Aromatics	10
R2	W5S	Nitrogen oxides	1
R3	W3C	Ammonia and aromatic molecules	10
R4	W6S	Hydrogen	100
R5	W5C	Methane, propane and aliphatic non-polar molecules	1
R6	W1S	Broad methane	100
R7	W1W	Sulfur-containing organics	1
R8	W2S	Broad alcohols	100
R9	W2W	Aromatics, sulfur-and chlorine-containing organics	1
R10	W3S	Methane and aliphatics	10

**Table 2:** Sensor response signal data for litchi in different storage environments.

Time	Storage Groups	R1	R2	R3	R4	R5	R6	R7	R8	R9	R10
0d	RT	0.313	2.448	0.486	1.103	0.542	6.422	630.558	4.936	3.316	1.214
	RE	0.394	2.263	0.574	1.089	0.633	4.491	583.561	3.660	2.759	1.114
	CA	0.351	2.305	0.521	1.388	0.580	5.057	552.293	3.876	2.740	1.109
2d	RT	0.241	4.377	0.384	1.121	0.433	9.337	2601.409	6.295	4.648	1.202
	RE	0.343	2.680	0.511	1.078	0.572	6.080	526.332	4.487	2.974	1.158
	CA	0.302	2.663	0.473	1.169	0.535	6.347	355.419	5.06	3.129	1.168
4d	RT	0.166	13.531	0.262	1.154	0.286	12.347	5873.842	7.487	17.022	1.184
	RE	0.328	2.739	0.476	1.064	0.536	5.641	389.635	4.185	3.143	1.116
	CA	0.273	2.738	0.401	1.152	0.468	6.632	75.887	4.754	3.206	1.100

Note: RT is the room temperature environment storage group, RE is the refrigerator environment storage group, and CA is the controlled-atmosphere storage group.

**Table 3:** BPNN classification results of storage times of litchi stored in three environments.

Storage Groups	Input Layers	Hidden Layer	Output Layers	Learning Factor	Dynamic Factor	Training Times	Accuracy/%	
							Training Set	Test Set
RT	10	19	5	0.05	0.85	20,000	89.33	52
RE	10	21	5	0.025	0.75	20,000	100	88
CA	10	18	5	0.05	0.85	20,000	100	96

Note: RT is the room temperature environment storage group, RE is the refrigerator environment storage group, and CA is the controlled-atmosphere storage group.

**Table 4:** Training parameters of BPNN hardness prediction models for different storage environments.

Storage Groups	Input Layers	Hidden Layer	Output Layers	Learning Factor	Dynamic Factor	Training Times	
RT	10	23	75	0.05	0.87	20,000	
RE	10	25	75	0.055	0.87	20,000	
CA	10	23	75	0.045	0.80	20,000	
GL	10	25	75	0.43	0.87	20,000	

Note: RT is the room temperature environment storage group, RE is the refrigerator environment storage group, CA is the controlled-atmosphere storage group, and GL is the global environment storage group.
